# Stress Concentration Factors in Excavation Repairs of Surface Defects in Forgings and Castings

**DOI:** 10.3390/ma15051705

**Published:** 2022-02-24

**Authors:** Alessandro Rebora, Giorgio Torre, Gianluca Vernassa

**Affiliations:** 1 Department of Mechanical Engineering, University of Genoa, Polytechnic School, Via All’ Opera Pia 15A, 16145 Genoa, Italy; rebora@unige.it (A.R.); s4256627@studenti.unige.it (G.V.); 2 SBM Offshore, 11 Avenue Albert II, 98000 Monaco, Monaco

**Keywords:** defects, stress concentration, repair, excavation, FEM

## Abstract

This paper provides an analytical formula for the theoretical stress concentration factor in a common type of excavation repair for large forgings and castings. Mechanical components obtained with these processes are often subjected to superficial defects. As the rejection of such pieces is out of question, given the relevant size and costs associated with them, usual industrial practice consists in the removal of the defect and a portion of the surrounding material through milling processes. The authors have selected a reference geometry of the excavation to be left on the mechanical pieces, which can be easily controllable in practice by three operating parameters. Then, the domain of existence of such a repair was investigated on a sequence of discrete points, by means of FEA, obtaining for each, the values of the stress concentration factor K_t_. Finally, through polynomial regression, the K_t_ functions have been accurately approximated by a sixth degree polynomial formulation, which, given a triplet of dimensional geometric parameters, is able to compute the stress concentration factor K_t,_ with an error that never exceeds 8%.

## 1. Introduction

Large mechanical components obtained by forging or casting can often be affected by different types of superficial defects. The most common are bubbles and cracks [1], which, depending on their shape and size, may lead to relevant stress concentration, compromising the long-term life of the part. To overcome this issue, one of the most common industrial practices consists of the simple technique of removing the defects and a portion of the surrounding material, through manual milling operations, made with a conventional disc cutter or with a ball nose cutter. Sometimes, after milling, the emptiness left on the surface is also welded, aiming to fill it completely with additive metal. However, not all ferrous materials may be easily subjected to this treatment. In addition, the time-consuming welding operations may have a negative impact on final delivery times. In all such cases, the remedy of sole milling does not bring the defective part to its ideal shape, since a small portion of the material is still removed from its surface, but leaves an imperfection of a more controlled shape. The stress amplification, therefore, persists, but at lower intensities and can be grasped quantitatively through accurate FEM analyses. However, these require the definition of numerical models for the specific purpose, where the detailed shape of the new defect must be added to the original complexity of the system. Models usually turn out to be very heavy computationally and the results are always a compromise between accuracy and time. A practical solution of engineering interest is the identification of a theoretical stress concentration factor K_t_, defined as the following ratio:

(1) $$K_{t}=\frac{\sigma_{\max}}{\sigma_{n}}$$

where $\sigma_{n}$ is the nominal stress (stress intensity that would develop in the region of interest, when the part is subjected to certain boundary conditions, if the geometrical discontinuity was not present) and $\sigma_{\max}$ is the maximum stress that develops due to the presence of the imperfection. It should be noticed that the definition of a theoretical K_t_ factor implies both linear elastic material behaviour and small displacements. For these reasons, the theoretical K_t_ is also known as “form factor”, or as “geometrical stress concentration factor” [2], since its value depends only on the geometrical form of the local imperfection causing the stress concentration effect and does not depend on the material Young’s modulus [3].

Once the excavation repair is applied on the defective part, a K_t_ factor can be calculated, $\sigma_{n}$ retrieved from the previous dimensioning calculations and finally, $\sigma_{\max}$ can be estimated. To our knowledge, the available technical literature does not provide general analytical formulas to calculate the numerical value of the K_t_ factor for this type of stress concentrator. To fill this need, a wide campaign of FEM simulations was organized and several K_t_ values were drawn from it. Then, through multiple regression operations, a suitable polynomial approximation of this stress concentration factor was obtained.

The shape of the portion of material removed from the defective piece depends on many geometrical and technological parameters [4,5,6,7]. Some can be controlled with great accuracy, while others embody the intrinsic variability of manual technological processes. When a ball nose cutter is manually used to repair the defect, the only geometrical parameter involved is the diameter of the ball nose. If instead, a disc cutter is used, the geometrical parameters to be considered are the diameter of the disc, its thickness and the fillets’ radius on its two circumferential edges. Although a small consumption of the cutting surfaces occurs during operation, geometrical parameters may be considered constant during the time of a single repair. The technological parameters, which are the ones more affected by uncertainties, are the depth of penetration of the tool, the inclination of incidence of its rotation axis and the pattern to be followed during the strokes.

In light of the difficulties in controlling all of these parameters with great accuracy during the industrial practices, a parametric numerical study that considers all the possible combinations would be worthless. Therefore, the authors have selected a simple reference shape of the excavation, as well as a set of the most relevant and easily controllable parameters, which aim to be considered in the future as a guideline for this type of repair.

## 2. Materials and Methods

**Figure 1:**
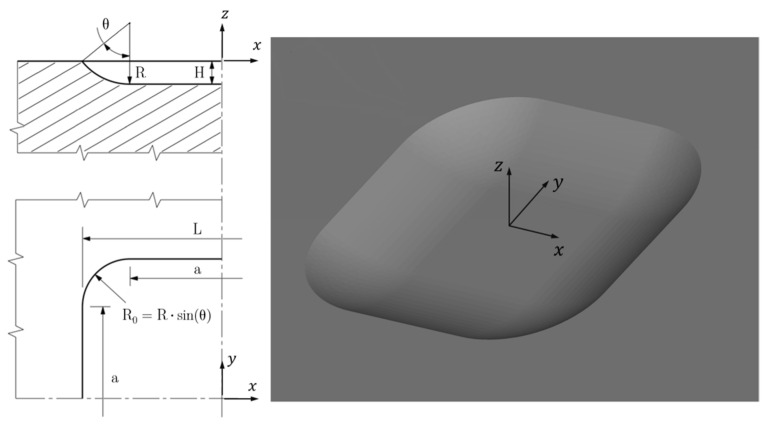
Quoted drawing and axonometric view of the square excavation.

The simplest reference shape that can be associated with the volume of material removed is the one of a square excavation (Figure 1) deep “H” and having, on the plane of the surface of the mechanical piece, the length of its side equal to “L”. On the bottom plane of the excavation, the length of the side is equal to “a”, being “a” < “L”, always. Such a shape of removed material can be obtained using a ball nose cutter of radius “R”, which initially penetrates the surface of the piece of a depth “H” and then moves consecutively along two orthogonal axes parallel to this surface. In this case the length of every milling stroke is equal to “a”.

As this type of repair interests the surface of the mechanical pieces, it is assumed that the nominal stresses $\sigma_{n}$ that the users shall retrieve are those characterizing a plane stress state, where in the general case two normal and one tangential stress components exist. In order to develop such a stress state in the numerical models employed in this study, the generic “perfect” mechanical piece, i.e., exempt from any geometric defect, has been modelled as an elastic half-space of theoretically “semi-infinite” extension. Practically, the characteristic dimensions of the elastic substrate have been chosen sufficiently large to satisfy the Saint-Venant principle, as set out in [8], by virtue of which, at that distance from the geometric imperfection, the perturbation of the plane stress state due to the defect is contained below a certain threshold.

As shown in Figure 1, the four side walls of the excavation are not vertical (i.e., parallel to the z axis) and are not flat surfaces, but are inclined cylindrical surfaces whose single curvature is equal to 1/R. These four curved surfaces are tangent to the flat bottom of the excavation, so that no geometric discontinuities are formed. The four edges of the excavation on the outer surface are joined together by four arcs of circumference of radius R_0_ < R, extending over an angle of 90°. It follows the presence of four double-curvature surfaces at the four excavation vertices, similar to triangular “sails”, that are all portions of the same spherical surface of radius R. By varying the value of the geometric parameters listed above (L, H, a, R) it is quite easy to adapt this general “reference” geometry to cover a wide collection of actual analysis cases to be studied by FEM calculations.

**Figure 2:**
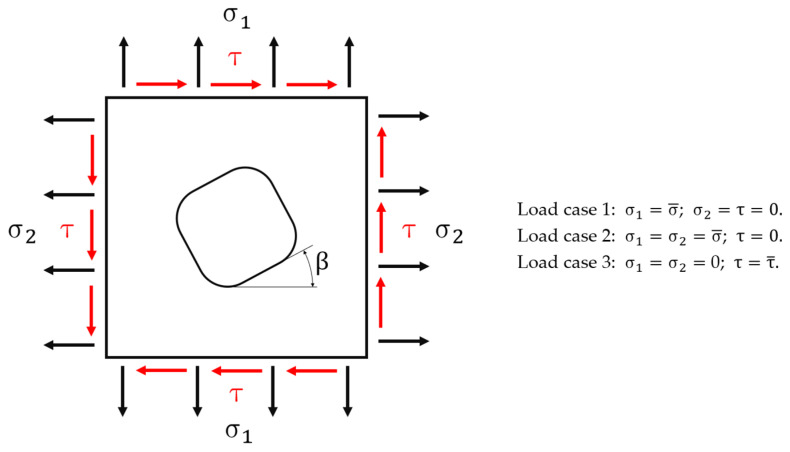
Loading conditions applied to the models’ sides.

Simple stress states to be applied to all FEM models are shown in Figure 2, which can be described as: (1) a uniaxial tensile load acting in a direction parallel to one of the sides of the excavation; (2) an equi-biaxial tensile load in the two directions parallel to the two sides of the excavation; (3) a pure shear stress.

In all the analyses run throughout this study the angle β has been assumed equal to 0°. This makes geometric and loading conditions such that only a quarter of the geometry needs to be modelled; solicitations are applied either perpendicularly, in load cases 1–2, or parallelly to the models’ sides, in load case 3. Moreover, symmetry boundary conditions, in load cases 1–2, and antisymmetry boundary conditions, in load case 3, must be applied to the nodes lying on the two cutting planes. However, these three different loading cases imply three different definitions of the stress concentration factor K_t_. In the first case, the meaning of K_t_ is the one originally defined by Kirsch [9], K_t_ = $\sigma_{\max}$**/**$\sigma_{n}$, where $\sigma_{\max}$ stands for the maximum peak uniaxial stress while $\sigma_{n}$ is the so called “nominal stress”. In the second case, the stress state is no longer uniaxial and therefore an equivalent Von Mises ideal stress must replace both previous stresses in order to define a new K_t_ = ${\left(\sigma_{id\;V.M.}\right)}_{\max}$**/**${\left(\sigma_{id\;V.M.}\right)}_{n}$, as proposed in [3,10]. In the third case, the stress state is still biaxial, but in such circumstances a more convenient expression of the stress concentration factor is then: K_t_ = $\tau_{\max}$**/**$\tau_{n}$ [2,11].

All FEM calculations have been executed by using the general-purpose code ANSYS APDL, rev. 2019 [12], obtaining, as a result of every sequential run, three different K_t_ values (K_t1_, K_t2_, K_t3_), valid for the first, second and third load cases, respectively. Afterwards, for every load case, a response−surface of K_t_ values was analysed and suitably approximated by a polynomial function of two independent dimensionless parameters.

Among the four geometric dimensional parameters listed above (L, H, a and R) only three of them are mutually independent, since a geometric relationship subsists, expressed by the two following equations:

(2) $$R\sin\theta =\frac{L-a}{2}$$

(3) R(1−cosθ)= H

To carry out a cyclic sequence of numerical finite element analyses, a single versatile parametric model can be employed. One of the three parameters can be referred to as the “reference length” and set to a constant value. In fact, the geometric shape of the excavation depends only on the ratio between the three independent dimensional parameters and not on their individual values. In this case, the value L was chosen to be kept constant.

The other two parameters then no longer assume the values of the individual lengths, but their ratios with the reference length, becoming hence dimensionless parameters. In our case these have been referred to as p_1_ and p_2_, where p_1_ = a/L and p_2_ = H/L.

The first limiting condition 0 < p_1_ < 1 comes from the already mentioned inequality “a” < “L”. The second one is assigned instead to the θ angle, which must vary in the open interval 0 < θ < π/2, as we assume that the ball nose cutter will never penetrate a depth H greater than half its diameter.

The result of this campaign of numerical calculations consists of the function of two variables K_t_ = K_t_ (p_1_, p_2_), which are obtained by points varying p_1_ and p_2_ in a discrete way. For this goal, it is necessary to assign the upper and lower boundary values for the dimensionless parameters p_1_ and p_2_, i.e., to set the limits of the two intervals:

(p_1_)_min_ ≤ p_1_ ≤ (p_1_)_max_ (4)

(p_2_)_min_ ≤ p_2_ ≤ (p_2_)_max_ (5)

The choice of the two minimum limits is very simple; in fact, it is possible to fix them arbitrarily by choosing, for example, (p_1_)_min_ = (p_2_)_min_ = 0.01. In particular, the first relationship a/L = p_1_ ≥ (p_1_)_min_ = 0.01 prevents the modelling of excavations too close to the limit shape of a spherical cap, that requires a particular FEM mesh, not obtainable through the analysed parametric FEM model by simply fixing a/L = 0. The second relationship H/L = p_2_ ≥ (p_2_)_min_ = 0.01 prevents the modelling of the almost imperceptible excavations in which the depth of penetration of the tool tends to vanish, as the volume of the removed material looks like a thin “flake”.

As regards the two maximum limits, it can be observed instead that the choice of (p_1_) conditions that of (p_2_) and vice versa. In fact, there are two linear mathematical laws:

(p_1_) = 1 − 2 (p_2_) (6)

(p_2_) = −0.5 (p_1_) + 0.5 (7)

which are a direct consequence of the two inequalities 0 < θ < π/2.

In any case, even the two maximum limits cannot reach the two respective extreme values (p_1_)_max_ = 1 and (p_2_)_max_ = 1/2. In fact, the condition a/L = 1 would give birth to a perfectly rectangular excavation, without any curved surface connecting its sides. On the other hand, the condition H/L ≥ 1/2 would correspond to an excavation with flat and vertical side walls, which could only be obtained if the cutter disc penetrated to a depth H greater than one half of its diameter.

In light of all the previous considerations, the limits have been decided as:

0.01 ≤ p_1_ ≤ 0.98 (8)

0.01 ≤ p_2_ ≤ 0.49 (9)

The two inequalities (8) and (9) appear to define a domain of definition of the function K_t_ = K_t_ (p_1_, p_2_) of a rectangular type. However, Equations (6) and (7) cut this domain along its descending diagonal into two triangular parts. In the lower triangular part, placed to the left of the matrix diagonal, the function exists. In the upper triangular part, placed to the right of the diagonal matrix, the function cannot exist, due to the inequality θ < π/2. For the numerical computations, both the intervals for p_1_ and p_2_ are divided into 43 parts. However, these discrete values are not equally spaced with each other, but thickened on the two extremes of the interval, where the K_t_ surface is more curved. The result is a square matrix of several points K_t_ = K_t_ (p_1_, p_2_).

The sequential execution of 44 × 44 = 1936 FEM analyses, of which only about one-half are actually carried out, provides all the calculation points on which to set up a polynomial regression. From an operational point of view, it is possible to perform all the FEM calculations in an uninterrupted way. In this way analyses are carried out one just after the other, without the waste of the dead time in between for the “manual” start-up of the code. This can be obtained by launching the structural analysis of a single parametric model written in ANSYS APDL language which is controlled, in its basic structure, by two mutually nested *DO cycles. The outermost *DO cycle governs the increase in the a/L parameter and determines the sequence of the rows of the K_t_ matrix. The innermost *DO cycle governs the increase in the H/L parameter and determines the sequence of the columns of the K_t_ matrix.

**Figure 3:**
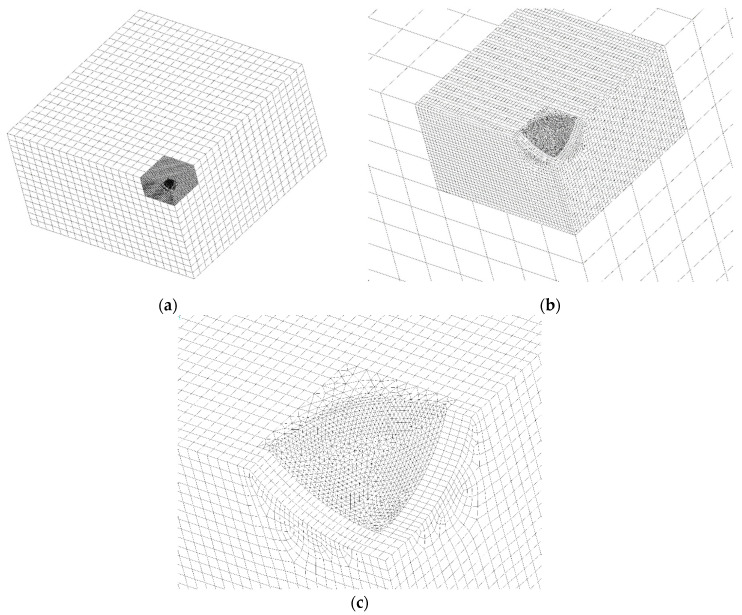
Three zoom views of the FEM model mesh. (**a**) Coarse overall mesh. (**b**) Finer central irregular mesh. (**c**) Pyramidal mesh generated under the triangular sail.

Figure 3a–c show, as an example valid for a/L = H/L = 0.20, the FEM model mesh composed of hexahedral second order 20-node brick elements, SOLID186 of ANSYS Element Library. The total number of elements is not constant, since among the models cyclically analysed it varies from a minimum of about 10^5^, to a maximum of about 3 × 10^5^.

In Figure 3a the coarse overall mesh surrounding the finer central mesh is a mapped and regular 3-D mesh of parallelepiped brick elements. Solicitations are applied to the two vertical surfaces that in this figure appear to be “hidden”. Specific symmetry or antisymmetry boundary conditions must be imposed to the nodes lying onto the geometrical symmetry planes corresponding to the two vertical surfaces that in this figure appear to be in view.

The finer central mesh of Figure 3b,c, instead, is not completely a mapped mesh, since near the zone of the curved excavation the element conformation is that of a pyramid with a rectangular base. The nodes in the FEM meshes of the two different regions are tied together by means of proper constraint equations, automatically generated by the ANSYS pre-processing command “CEINT”.

The further zoomed view of Figure 3c shows some details of the pyramidal mesh generated under the triangular sail, where the maximum K_t_ always occurs.

The mesh quality has been investigated by using the criterion of the strain energy error [13,14], already applied in [15]. In all the analysed FEM models the value of the structural percentage error in energy norm (SEPC) is never greater than 1.5%. This value is under the conventional limit that addresses the quality of mesh in the local area of high stress. This is a sign of an optimal mesh size in all analysed cases.

## 3. Results

**Figure 4:**
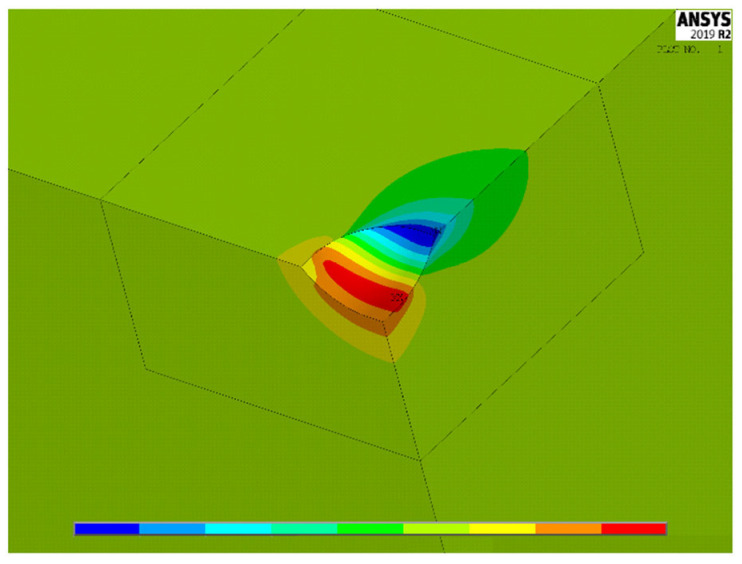
Distribution of K_t_ values in load case 1.

**Figure 5:**
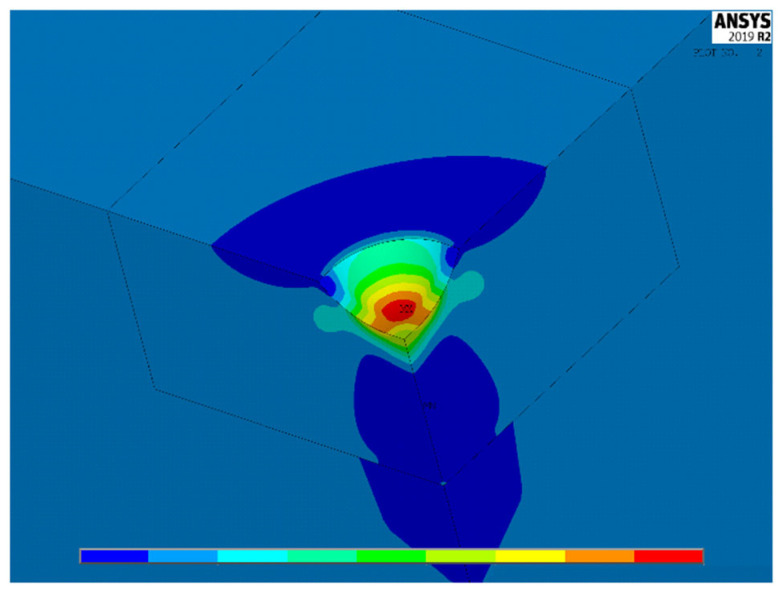
Distribution of K_t_ values in load case 2.

**Figure 6:**
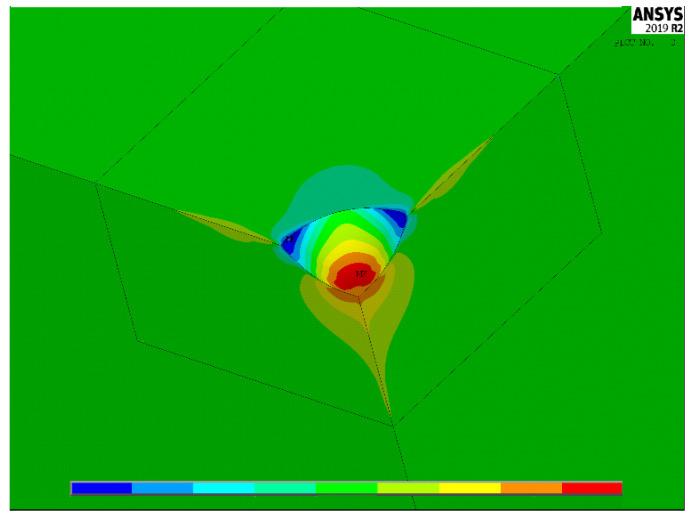
Distribution of K_t_ values in load case 3.

Figure 4, Figure 5 and Figure 6 show, for the same model as Figure 3, the contour lines plotting the distribution of the stress concentration factor K_t,_ calculated for the three load cases 1–3, respectively. They are intended to be simply qualitative images, without K_t_ values, here reported just to show the position where the maximum stress concentration occurs. As expected, the position of the point of maximum stress lies on a symmetry plane in load case 1, while, in load cases 2 and 3, it is found on a diagonal plane, bisecting the right angle between the two planes of symmetry, or antisymmetry.

**Figure 7:**
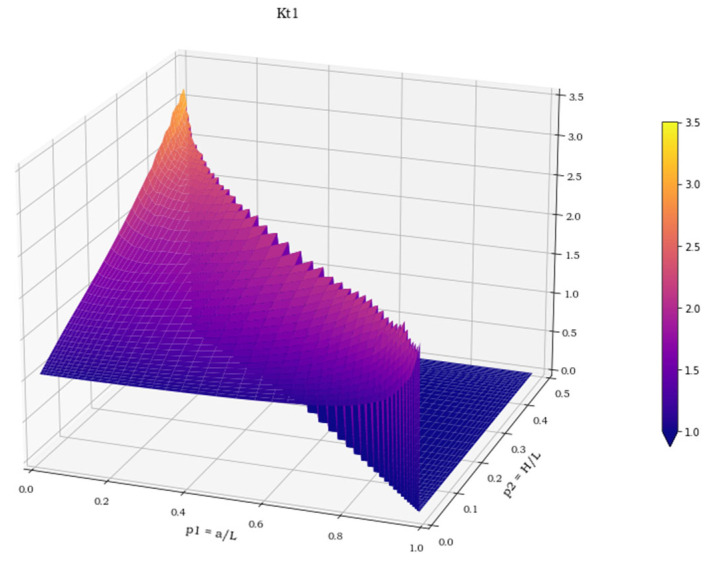
K**_t_** values in load case 1.

**Figure 8:**
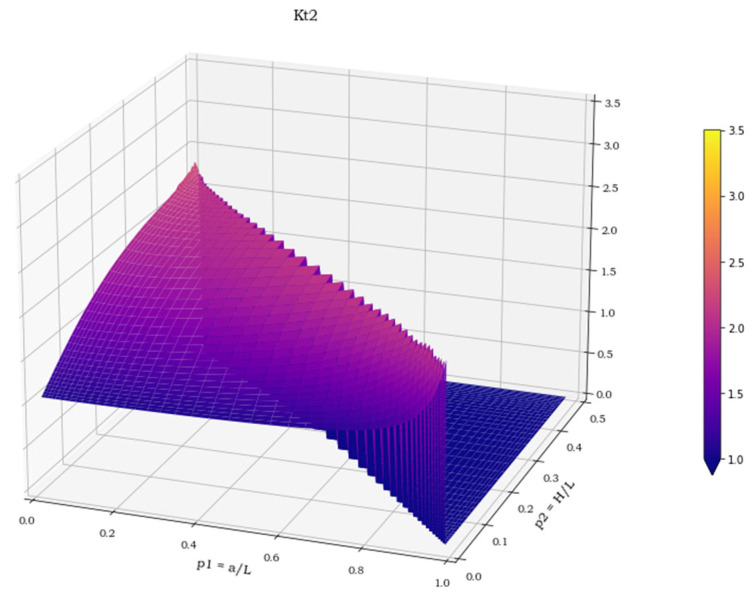
K**_t_** values in load case 2.

**Figure 9:**
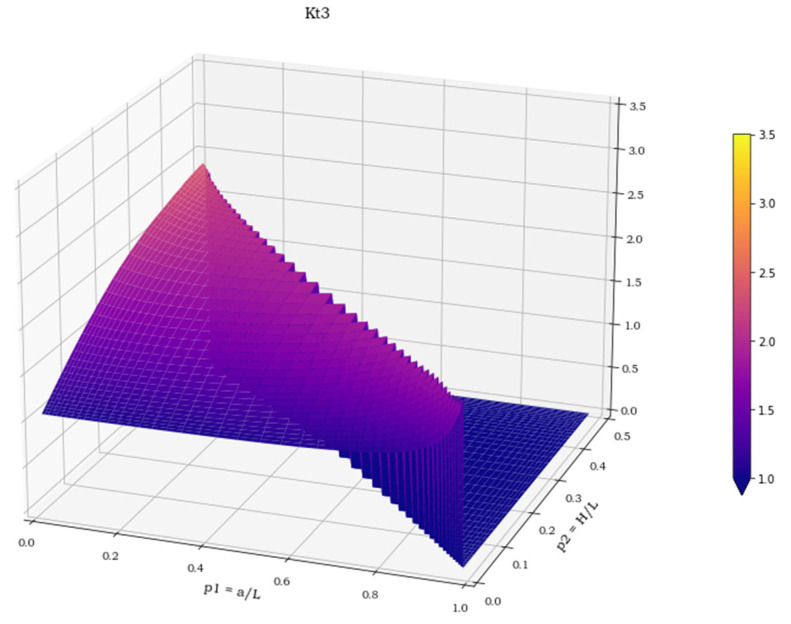
K**_t_** values in load case 3.

The three spatial surfaces, shown in Figure 7, Figure 8 and Figure 9, plot the calculated values of the three stress concentration factors K_t_ = K_t_ (p_1_, p_2_), obtained for load cases 1–3, respectively. Where the function does not exist, its value has been arbitrarily set to 0. The next section deals with the discussion of these implicit functions and illustrates the numerical methods employed to perform a suitable polynomial regression.

The numerical regression was applied to find the coefficients of a sixth-degree polynomial, written as:

(10) $$\begin{array}{ll} K_{tj} & =a_{0j}+a_{1j}p_{1}+a_{2j}p_{2}+a_{3j}p_{1}^{2}+a_{4j}p_{1}p_{2}+a_{5j}p_{2}^{2}+a_{6j}p_{1}^{3}+a_{7j}p_{1}^{2}p_{2}+a_{8j}p_{1}p_{2}^{2}+a_{9j}p_{2}^{3}+a_{10j}p_{1}^{4}+a_{11j}p_{1}^{3}p_{2}\\ & +a_{12j}p_{1}^{2}p_{2}^{2}+a_{13j}p_{1}p_{2}^{3}+a_{14j}p_{2}^{4}+a_{15j}p_{1}^{5}+a_{16j}p_{1}^{4}p_{2}+a_{17j}p_{1}^{3}p_{2}^{2}+a_{18j}p_{1}^{2}p_{2}^{3}+a_{19j}p_{1}p_{2}^{4}+a_{20j}p_{2}^{5}+a_{21j}p_{1}^{6}+\\ & +a_{22j}p_{1}^{5}p_{2}+a_{23j}p_{1}^{4}p_{2}^{2}+a_{24j}p_{1}^{3}p_{2}^{3}+a_{25j}p_{1}^{2}p_{2}^{4}+a_{26j}p_{1}p_{2}^{5}+a_{27j}p_{2}^{6}\;(j=1,3)\; \end{array}$$

where K_t1_, K_t2_, K_t3_ are the stress concentration factors calculated for the three load cases 1–3, respectively. Formula (10) is only valid under the conditions 0.01 ≤ p_1_ ≤ 0.98 and 0.01 ≤ p_2_ ≤ 0.5 − 0.5 p_1_.

The regression problem was solved using the least square method, implemented in the function linalg.lstsq, available in the Python library Numpy [16]. In Table 1, the (28 × 3) a_ij_ coefficients are reported.

## 4. Discussion

**Figure 10:**
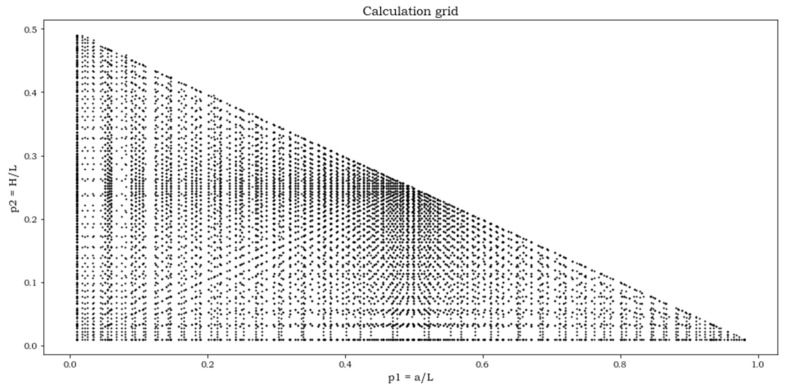
Grid representing FEA solution points.

The FEA solution, for the three load cases, has been obtained for each of the parameter combinations indicated by a dot in Figure 10. A total of 10,201 solutions were available for each load case.

**Figure 11:**
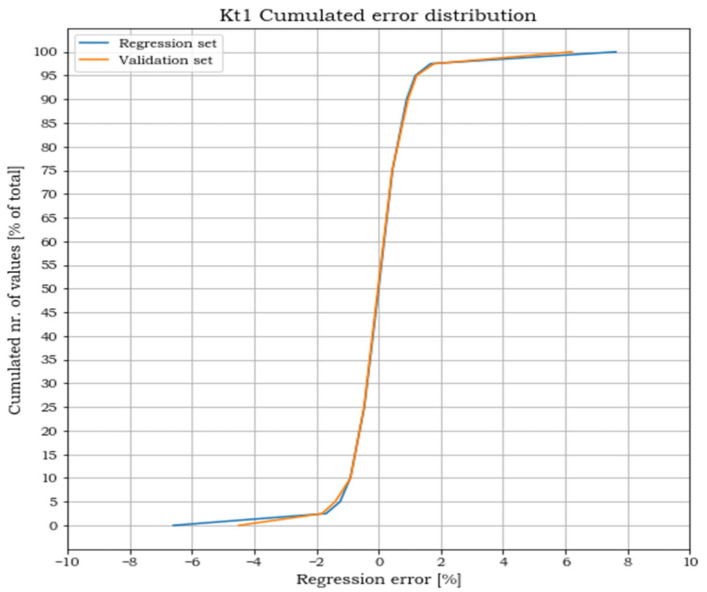
Cumulated error distribution for load case 1.

**Figure 12:**
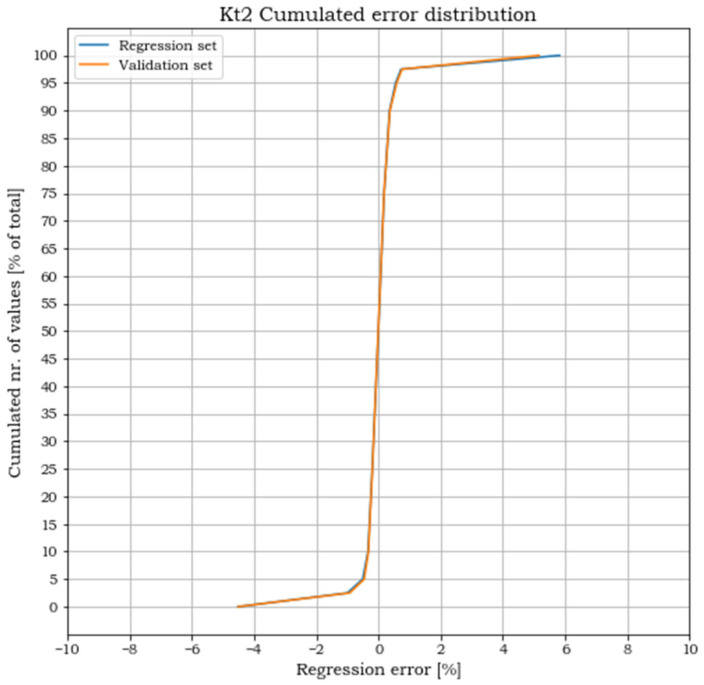
Cumulated error distribution for load case 2.

**Figure 13:**
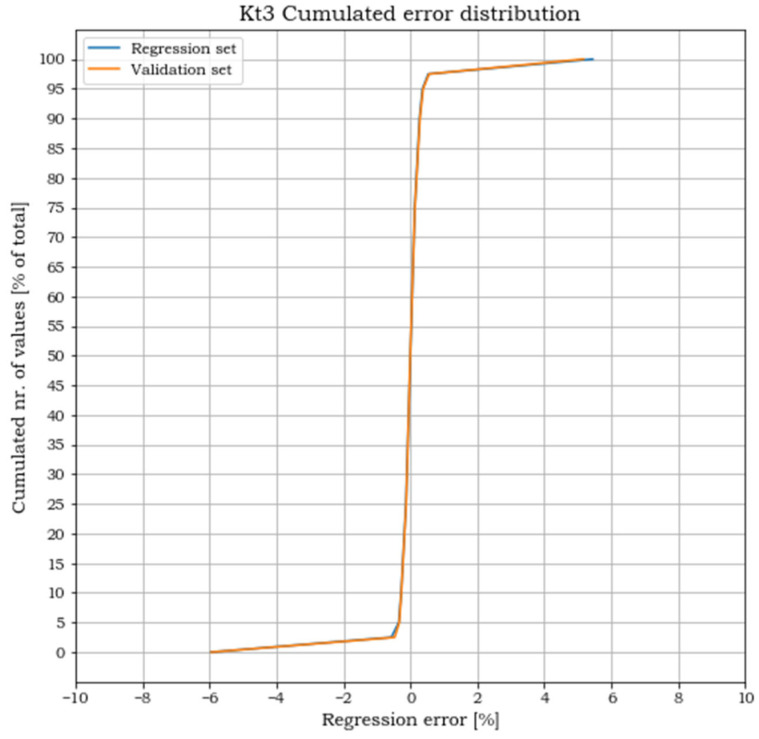
Cumulated error distribution for load case 3.

A subset of 5100 randomly selected points, later referenced as regression set, has been used for solving the regression. The remaining solution points, referenced instead as validation set, have been used to validate the effectiveness of the polynomial approximation in matching the FEA results (the separation of data between a regression set and a validation set is inspired to the split applied to data in the training of neural network models where the splits set are defined as training and validation set. In this case, the usage of a linear least square, which does not require an iterative process for the solution, such as those used in the training of neural networks, suggests the introduction of a different term, regression set, to remind of the different nature of the process). The validation consisted of two steps. The first step ensures that the error committed in the polynomial approximation of the K_tj_ is within acceptable limits. The second step ensures that the error committed in the evaluations of the points belonging to the validation set is of the same magnitude as that of the points in the regression set. The regression error is calculated, for each point i and for each load case j, as:

(11) $$e_{ij}=\frac{K_{t\_ij\_regr}-K_{t\_ij}}{K_{t\_ij}}$$

where $K_{t\_ij\_regr}$ and $K_{t\_ij}$ are respectively the stress concentration factors obtained from the polynomial regression and from the FEA. Plots of the cumulated number of points versus the corresponding error levels are shown in the following Figure 11, Figure 12 and Figure 13 for both the regression and the validation sets.

**Figure 14:**
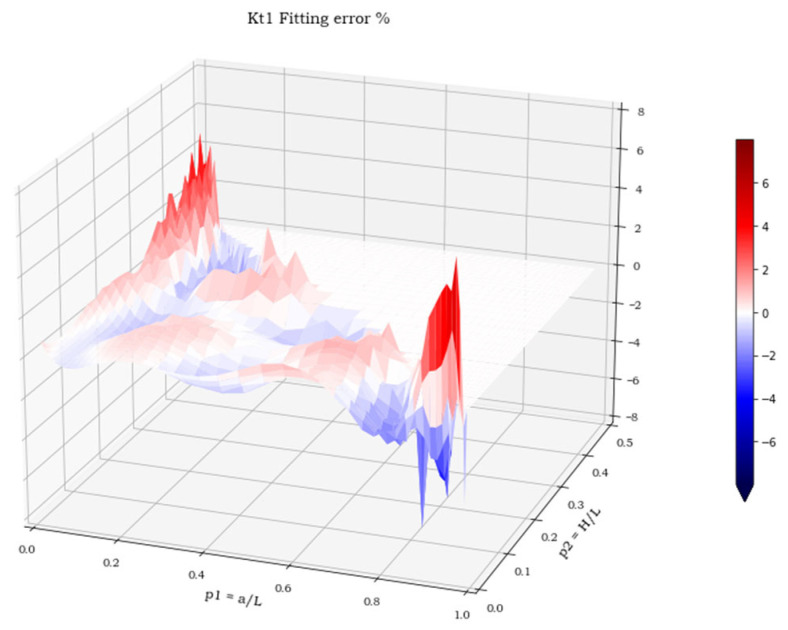
Regression error in load case 1.

**Figure 15:**
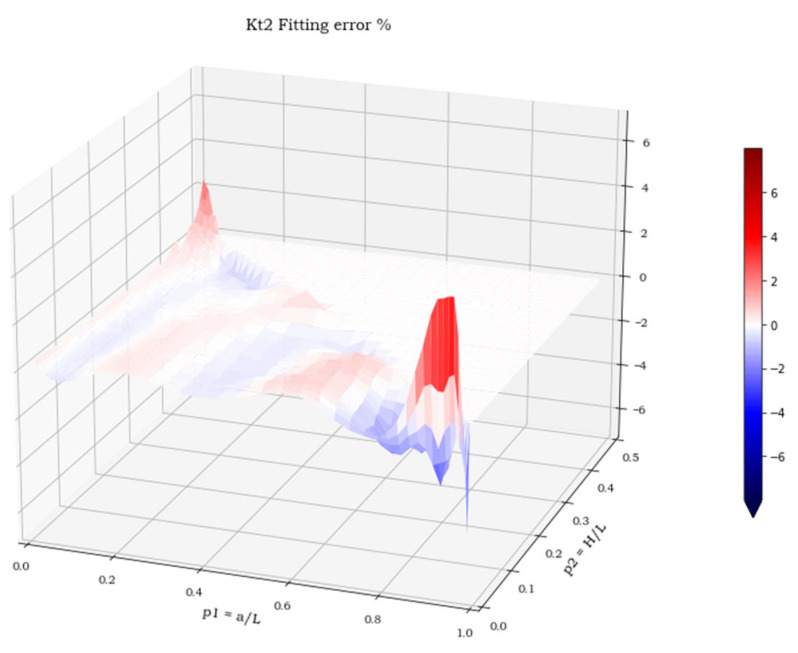
Regression error in load case 2.

**Figure 16:**
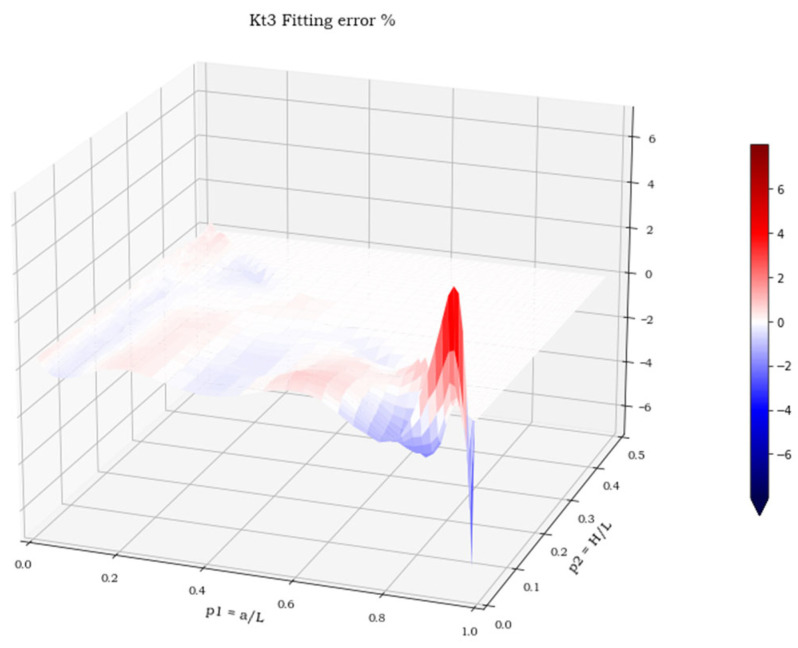
Regression error in load case 3.

For all three load cases, more than 90% of the points lay within an error band of ±2%. The cumulated plots for the regression and the validation sets are almost identical, which confirms the effectiveness of the fit for points outside the set used for the regression and the absence of overfitting. The error extremes are never greater than 8% and, as shown in Figure 14, Figure 15 and Figure 16, are localized where the pairs (p_1,_ p_2_) approach the two points (0, 0.5) or (1, 0).

## 5. Conclusions and Future Developments of Analysis

At the end of this research, we can conclude that the analytical expressions provide a suitable approximation of the stress concentration factor for this geometry under the three most common load case scenarios. Moreover, we can assert that a gap in the technical literature of the sector has been filled, as this type of excavation, obtained by means of a ball-headed cutter, had not been investigated yet in terms of stress concentration. Finally, our analyses have ascertained that load case 1 (uniaxial tensile load acting in a direction parallel to one of the excavation’s sides) is the most dangerous, as it reaches the higher K_t_ values.

The real novelty that characterizes this publication certainly does not lie in the method of investigation, which has already been applied by numerous other authors [2,10,11], in the same form in which it was applied here. In fact, the systematic generation of large amounts of numerical data, i.e., the K_t_ values, as a function of one or more independent parameters, in this case the a/L and H/L ratios, and finally, the re-elaboration of the same, with conventional regression techniques, is certainly a well-known methodology to all researchers active in the sector.

However, it is the particular geometric shape of the stress concentrator, i.e., the square excavation, which, despite being relatively simple to be made practically on the defective pieces being repaired, is instead very complex to control correctly, through few independent geometric parameters and, therefore, it had never been studied until today.

The difficulties in generating the geometric shape of this model then become critical for the two “degenerate” shapes of the removed material, i.e., the one tending to the spherical cap and the other tending to a very thin flake of a square shape, whose respective FEM models must, however, always be adequate, that is respectful of the criterion of strain energy error.

Furthermore, although the use of full 3-D FEM models, i.e., composed of all second order solid elements, is not an important novelty to underline, both the total number of analyses performed (more than 10,000) and the average number of elements components of the generic FEM model (variable between 10^5^ and 3 × 10^5^) are numbers not easily found in the works of other authors. This is the necessary premise to an excellent accuracy of the results obtained in this work.

Finally, it should be emphasized that the numerical regression, carried out here according to the least squares criterion, was also addressed by the authors through the use of artificial intelligence algorithms, obtaining very similar results to those published here, albeit with a greater expenditure of time for preparation of post-processing analyses. For this reason, the paper did not mention artificial intelligence algorithms, which are moreover very promising, due to their extreme versatility and are, therefore, fit for purpose in more complex cases than this one.

From the examination of the obtained results, some guidelines and some suggestions emerge, which can already be understood with the simple common sense of the expert designer, very useful for planning this type of repair in the best possible way. In the case of a crack, with a depth greater than its surface extension, if the objective to be achieved is the minimization of the volume of material removed, the ideal shape of the excavation is the hemispherical one, despite the worst a/L and H/L ratios. On the contrary, there is the minimum perturbation of the stress state (and therefore the lowest K_t_), when the depth of the excavation becomes small if compared to its longitudinal dimensions, that is to say, if H/L tends to zero. It is also clear that the final decision on the type of intervention also depends on other factors that cannot be controlled in the study here addressed, such as the actual availability of the tools with the required dimensions or the proximity of the defect to structural details, such as spokes or stiffeners, that cannot be weakened by too wide a removal of material.

However, our work can be extended and further developments can be expected. Further research could concern the angle between the excavation edges and the line of action of the external forces. In fact, the geometry herein analysed only considered the case of parallelism between the two pairs of axes, assuming a relative rotation β equal to 0°. Although, all the possible intermediate angular positions included in the range 0° < β < 45° should be analysed, since they are expected to provide different and maybe higher results. Another development that could be envisaged originates from the study of more generalized geometries for the repair excavation. In this study, a square geometry with only one value for the fillet radii is studied. In a more general approach, excavations of rectangular shapes and with different filled radii could be studied. Such geometry would increase the number of parameters used to uniquely describe the problem. One of the consequences would be an increase in the parameter combinations to cover the design space and, consequently, of the number of FEA solutions needed to build an approximated formula. Another consequence would be, because of the increased dimensionality, the impossibility of directly plotting the stress concentration factor surfaces and the error committed in the polynomial approximation, as a function of all the problem variables. The formal evaluation of the effectiveness of the fit will, thus, have to rely exclusively on the similitude between the cumulated error plots of the regression and of the validation sets.

## Figures and Tables

**Table 1:** The a_ij_ coefficient matrix.

Coeff	Load Case 1	Load Case 2	Load Case 3
a_0_	1.016961394 × 10^0^	1.006965992 × 10^0^	1.007242102 × 10^0^
a_1_	−7.714813786 × 10^−1^	−4.874091070 × 10^−1^	−4.709116740 × 10^−1^
a_2_	3.730781538 × 10^0^	5.261191914 × 10^0^	4.887597416 × 10^0^
a_3_	8.541540052 × 10^0^	6.325601734 × 10^0^	6.099817374 × 10^0^
a_4_	9.243997472 × 10^0^	−2.354852538 × 10^0^	−2.369479447 × 10^0^
a_5_	1.708568460 × 10^0^	−4.225881840 × 10^0^	−2.798267490 × 10^0^
a_6_	−4.016474714 × 10^1^	−3.243507132 × 10^1^	−3.103958924 × 10^1^
a_7_	−2.625554101 × 10^1^	3.358136920 × 10^1^	1.965519297 × 10^1^
a_8_	−1.433009292 × 10^2^	−1.728446950 × 10^1^	−2.791846174 × 10^1^
a_9_	−2.098923527 × 10^1^	−3.023807318 × 10^0^	6.666379969 × 10^0^
a_10_	9.369387507 × 10^1^	7.803176090 × 10^1^	7.365966277 × 10^1^
a_11_	−4.609789596 × 10^1^	−1.101709869 × 10^2^	−6.636850028 × 10^1^
a_12_	9.943556248 × 10^2^	1.452036523 × 10^2^	1.457329063 × 10^2^
a_13_	1.073163653 × 10^2^	−3.526684769 × 10^0^	7.598207310 × 10^0^
a_14_	6.017300831 × 10^1^	−4.697832157 × 10^1^	−7.769518307 × 10^1^
a_15_	−1.046027393 × 10^2^	−8.824244625 × 10^1^	−8.172884217 × 10^1^
a_16_	2.008368360 × 10^2^	1.972152159 × 10^2^	1.172865395 × 10^2^
a_17_	−1.306438606 × 10^3^	−1.475331578 × 10^2^	−1.268687641 × 10^2^
a_18_	−2.699040278 × 10^3^	−6.902640918 × 10^2^	−5.143138024 × 10^2^
a_19_	7.028675237 × 10^2^	2.778015594 × 10^2^	2.369568558 × 10^2^
a_20_	−3.021019396 × 10^0^	1.570588351 × 10^2^	1.830760539 × 10^2^
a_21_	4.419178527 × 10^1^	3.771807048 × 10^1^	3.415978402 × 10^1^
a_22_	−1.119996354 × 10^2^	−9.156577133 × 10^1^	−4.771804624 × 10^1^
a_23_	3.136686513 × 10^2^	−1.869409980 × 10^2^	−1.244095291 × 10^2^
a_24_	2.252885456 × 10^3^	7.613198334 × 10^2^	5.235969502 × 10^2^
a_25_	2.306621887 × 10^3^	5.178864939 × 10^2^	3.256355455 × 10^2^
a_26_	−1.189832390 × 10^3^	−4.146839531 × 10^2^	−3.185600009 × 10^2^
a_27_	−7.391816479 × 10^1^	−1.197524163 × 10^2^	−1.310156588 × 10^2^

## Data Availability

Data are contained within the article.
